# Preoperative assessment and treatment of appendiceal mucocele complicated by acute torsion: a case report

**DOI:** 10.1186/1756-0500-7-1

**Published:** 2014-01-02

**Authors:** Christoffer Stark, Mikko Jousi, Berndt Enholm

**Affiliations:** 1 Department of Surgery, Päijät-Häme Central Hospital, Keskussairaalankatu 7, 15850 Lahti, Finland; 2 Department of Radiology, Päijät-Häme Central Hospital, Keskussairaalankatu 7, 15850 Lahti, Finland; 3 Department of Surgery, Turku University Hospital, Kiinamyllynkatu 4-8, 20520 Turku, Finland

**Keywords:** Appendix, Mucocele, Torsion, Ultrasonography, Magnetic resonance imaging, Appendectomy, Laparoscopy

## Abstract

**Background:**

Mucus-producing tumours of the appendix or mucoceles can, if left untreated, lead to dissemination of its contents into the peritoneal cavity causing substantial morbidity to the patient. Symptoms for complicated mucoceles can mimic those of acute appendicitis and the final diagnosis is most likely made intraoperatively. We here present a case that is, to our knowledge, one of only ten described in the literature and the first to characterize torsion of an appendiceal mucocele with abdominal magnetic resonance imaging.

**Case presentation:**

The patient, a 34-year-old Caucasian female presented at the emergency department with acute abdominal pain in the right lower quadrant. Initial diagnostic work-up including ultrasonography and abdominal magnetic resonance imaging showed a large tubular mass at the base of the appendix with indirect signs of torsion. A laparoscopic appendectomy was performed the following day where the finding was confirmed. The patient went on to have an uneventful recovery and was discharged from the hospital on the first postoperative day.

**Conclusions:**

Magnetic resonance imaging is a useful tool in identifying unknown lesions of the appendix and should be considered the primary imaging modality in especially younger patients requiring diagnostic imaging. In this case the preoperative imaging findings aided in choosing the correct timing and treatment option for the patient.

## Background

The frequency of primary appendiceal tumours is reported to be 0.5-2% in removed specimens [1-4], mucoceles accounting for only 8% of them [5]. The epithelial lining of the appendix consists of abundant exocrine goblet cells, and thus most tumour types seen in appendiceal samples are mucus producing [6]. Excessive production of mucus by adenomatous tumours leads to the formation of a mucocele and is usually caused by entrapment of mucus and characterized by invasion of mucus into the appendiceal wall [2]. Mucoceles within the appendix are highly heterogenous upon histopathological evaluation and can be classified according to their underlying epithelial neoplastic processes, as proposed by Pai and Longacre [7]. Non-neoplastic or non-epithelial processes, such as inflammation or fecolith obstruction are rare causes of mucocele formation [7]. The majority of mucoceles are fortunately benign in nature [5,6]. Rupture of these tumors can however lead to dissemination of the abdominal cavity causing localized or generalized pseudomyxoma peritonei [2]. Thorough evaluation of suspicious appendices is therefore necessary in order to exclude rupture and avoid spillage of appendiceal contents into the abdominal cavity during appendectomy [6]. For mucoceles restricted to the appendix simple appendectomy either by open or laparoscopic technique is recommended [2,7]. Microscopic examination is recommended to ensure negative margins of resection surfaces [6,7]. Complications for appendiceal mucoceles (AM) consists of inflammation, invagination, obstruction, bleeding, or fistula formation [8]. Here, we present a rare case of AM complicated by acute torsion, characterized by abdominal magnetic resonance imaging (MRI). Preoperative diagnosis of this condition is difficult. Suspicion can however be valuable in choosing the correct timing and treatment option for the patient.

## Case presentation

**Figure 1:**
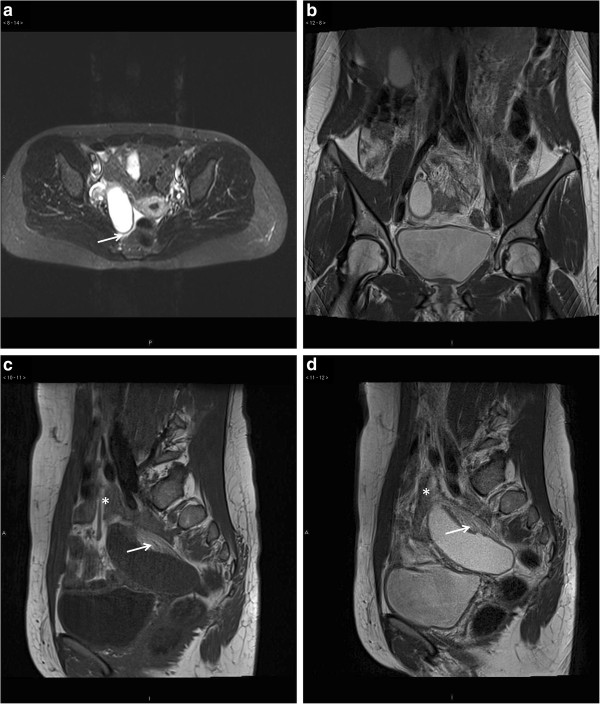
**Axial short T1 inverted recovery sequences (a) shows a regular appendiceal fluid-filled mass.** On T2-weighted sequences the mass is hyperintense (**b**, coronal view and **d**, sagittal view) and on T1-weighted sequences hypointense (**c**, sagittal view). The mesentery is edematic (**c** and **d**, white arrows), the base of the mucocele is indistinguishable from the appendix (**c** and **d**, *) and the mucocele is surrounded by fluid (**a**, white arrow) indicative of reactive inflammation and torsion.

**Figure 2:**
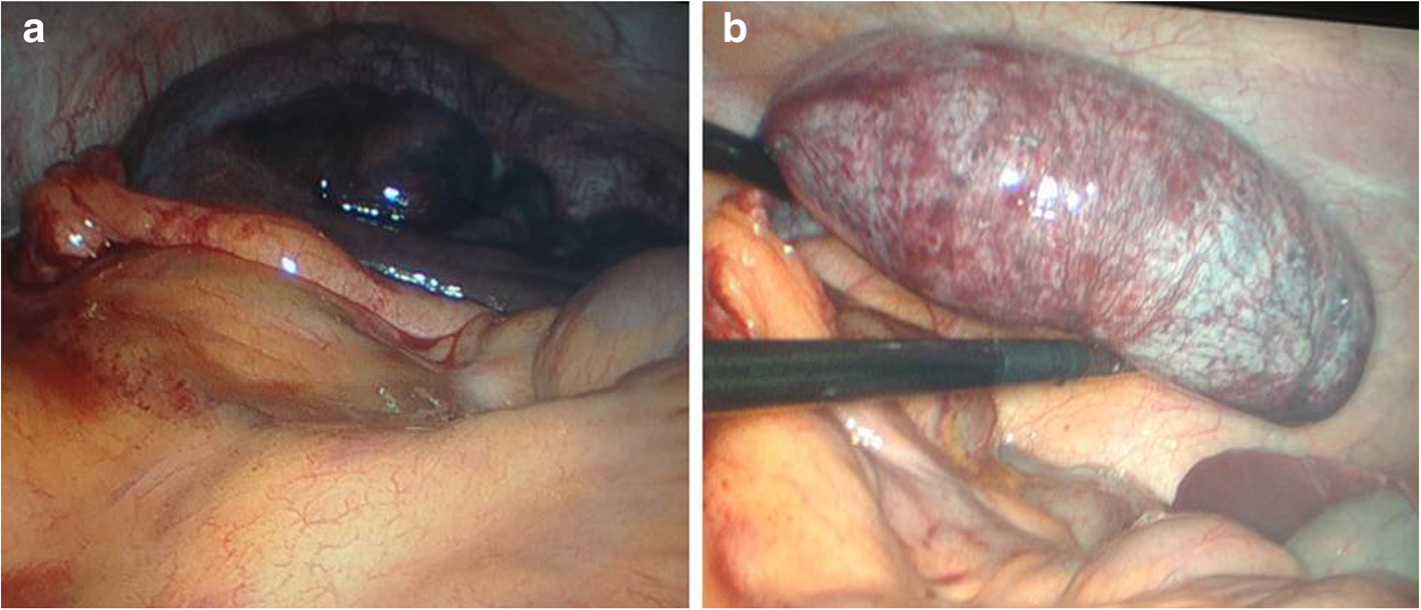
**Laparoscopic view of the appendiceal mucosele (a).** A clockwise 720 degree torsion can be seen at the base of the mucocele **(b)**. The appendix was gangrenous and hemorrhagic.

**Figure 3:**
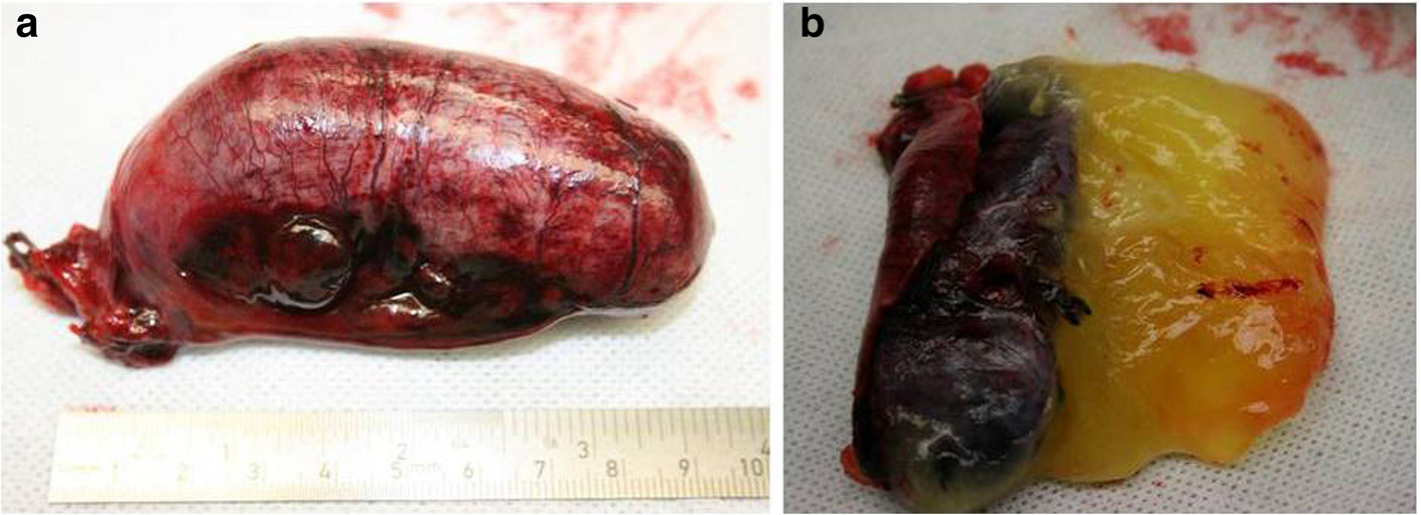
Removed appendix (a) displaying the mucous content (b).

A 34-year-old Caucasian female presented at the emergency department with an 8-hour history of nausea, vomiting and abdominal pain. The patient had suffered from occasional and transient episodes of abdominal discomfort during the last year. At the day of presentation the patient first experienced diffuse upper abdominal discomfort with later sudden pain in the right lower abdomen. The patient had palpable tenderness in the right lower quadrant, without signs of peritoneal irritation. The patient was afebrile and had a hemoglobin level of 145 g/l and a white blood cell count of 10.7 E9/l. The level of C-reactive protein was normal. Urine analysis was negative as well as analysis for human chorionic gonadotropin. The patient responded poorly to analgesic medications and a gynecological consultation was requested to exclude torsion of the ovary. Vaginal ultrasonography revealed a regular cystic mass between the uterus and the right ovary. This finding was verified with transabdominal ultrasonography, which showed a tubular mass of unknown origin. In order to characterize the finding further an MRI of the lower abdomen was performed. On MRI a fluid-filled appendiceal mass (8x3x3 cm) with relative wall-thickening was observed. The surrounding structures showed inflammatory changes with edema of the appendiceal mesentery. Abrupt tapering of the appendix was seen at the base of the mucocele (Figure 1). Based on the imaging results and acute clinical presentation a laparoscopic appendectomy was performed immediately the following working day. A distended and necrotic appendix was observed with a 720 degree clockwise torsion of its base (Figure 2). The abdominal cavity was otherwise macroscopically normal, with no signs of dissemination. Detorsion of the appendix was performed and the specimen removed using a bag according to normal operative protocol, taking care not to perforate the specimen. The appendix was cut open and a yellow gel-like content was observed (Figure 3). An additional resection of the base of the caecum was performed using a stapling device and surgical margins were confirmed by histopathological diagnosis. Frozen tissue sections of the mucocele showed necrosis of the appendicular wall making histological typing of the mass difficult, and the caecal resecate showed normal intestinal histology. The patient had an uneventful recovery and was discharged from the hospital the following day. The final pathological analysis showed distension of the appendix with flattening of the mucosa. Secondary necrosis and hemorrhage was observed in the appendicular wall with no signs of epithelial atypia. The muscular wall was not invaded by mucus or epithelium and the appendix was intact with no signs of mucus extravasation. The base of the caecum showed normal intestinal histology with no signs of mucus deposition or cellular atypia on permanent tissue sections.

## Discussion

Secondary torsion of the appendix due to a mucocele is rare, with only 10 previous case presentations in the English literature [8-14]. Preoperative assessment of this condition is challenging and the final diagnosis is usually established intraoperatively [9]. Sonographically mucoceles usually present as homogenous and hypoechogenic regular cystic lesions [13]. On computed tomography the changes are well circumscribed with Hounsfield values close to that of water. On MRI the lesions are hyperintense on T2-weighted sequences and hypo- or isointense on T1-weighted sequences [15,16]. Some characteristics of imaging findings in AM with torsion have been reported. Hebert *et al.* showed whorling of the appendiceal mesentery on computed tomography indicative of appendiceal torsion [12]. In another report by Hamada *et al.*, a cystic appendiceal mass showed a target-like appearance on ultrasonography and was later confirmed to present torsion of an AM [13]. Table 1 summarizes the main imaging findings in cases of torsion of appendiceal mucoceles found in the English literature.

**Table 1:** Findings and treatment in reported cases of torsion of appendiceal mucoceles with pre-operative imaging studies

**Age/gender**	**Modality**	**Imaging finding**	**Treatment**	**Reference**
34/F	US/MRI	Thin walled appendiceal cyst on US Fluid-filled, tubular appendiceal cyst with slight wall-thickening, mesentery edema and abrupt tapering of the baseof the mucocele on MRI	Laparoscopic appendectomy	Present
78/F	CT	Well encapsulated mass of unknown origin with intraluminal gas and slight calcifications	Open appendectomy	[10]
28/F	CT	Cystic thin-walled lesion with suspected ovarian cyst rupture	Laparoscopic appendectomy	[8]
34/M	CT	Appendiceal fluid filled cyst without contrast enhancement	Open appendectomy	[11]
59/M	CT	Dilated fluid-filled appendix negative for oral contrast, abrupt luminal tapering and whorled mesentery	Open appendectomy	[12]
79/M	US	Cystic appendiceal mass with target- like appearance	Open appendectomy	[13]
32/pregnant	US	Cystic lesion with internal echoes of unknown origin	Cesarean section and appendectomy	[14]

F = female, M = male, US = sonography, CT = computed tomography, MRI = magnetic resonance imaging.

In our case, the imaging findings were indicative of an appendiceal mucocele. The mesenteric edema and abrupt tapering of the base of the appendix were considered signs of torsion. MRI findings in AM with torsion have not previously been presented in the literature. Based on our findings, the discrimination between simple and complicated mucoceles by imaging is difficult. The patient’s sudden onset of abdominal symptoms and disease presentation were however inconsistent with classical appendicitis and the MRI findings aided in the pre-operative work-up of the patient. Notably, the appendicular wall had already developed necrosis highlighting the risk of rupture. Prompt laparoscopy provided the definitive macroscopic diagnosis and allowed for safe removal of the diseased appendix. Performing the procedure during office-hours ensured access to histopathologic analysis and confirmation that there was no neoplastic involvement of the caecum. The underlying pathology of the mucocele could however not be established on frozen sections. In cases where resection margins are negative and the muocele intact simple appendectomy is considered sufficient [6]. Frozen section analysis can therefore be helpful in cases of AM, although the final histopathological diagnosis requires permanent section analysis. Histologically the large mucocele was confined to the appendix and the luminal side of the intestinal wall with no signs of involvement of the serosa or surrounding compartments. In the necrotic and hemorrhagic specimen no epithelial atypia was observed. Permanent sections of the base of the caecum showed normal intestinal histology without signs of cellular atypia or mucus deposition. The underlying pathology was therefore thought to best represent a mucinous adenoma [7]. In this case simple appendectomy was curative with no need for additional treatments or long-term follow-up.

## Conclusions

It is the opinion of the authors that the threshold for performing imaging studies in patients with suspected appendicitis and atypical disease presentation should be low, in order to identify lesions that preferably should be operated on during daytime. To avoid complications and the need for reoperation in cases like the one presented here, the availability of senior surgeon and pathologist consultations during the operation is recommendable. Abdominal MRI is a useful tool in identifying unknown lesions of the appendix and should be considered the primary imaging modality in especially younger patients requiring diagnostic imaging when ultrasonography is inadequate.

## Consent

Written informed consent was obtained from the patient for publication of this Case Report and any accompanying images. A copy of the written consent is available for review by the Editor-in-Chief of this journal.

## Competing interests

The authors declare that they have no competing interests.

## Authors’ contributions

CS prepared the manuscript, and it was reviewed by MJ and BE. MJ analyzed the imaging results and prepared the MRI images. BE performed the appendectomy assisted by CS. All authors read and approved the final manuscript.

## Authors’ information

CS is a resident completing his basic surgical training at Päijät-Häme Central Hospital (PHCH) and a PhD student at the University of Turku. BE is a senior surgeon at PHCH. MJ is a consulting radiologist at PHCH.
